# Pyroptosis: mechanisms and diseases

**DOI:** 10.1038/s41392-021-00507-5

**Published:** 2021-03-29

**Authors:** Pian Yu, Xu Zhang, Nian Liu, Ling Tang, Cong Peng, Xiang Chen

**Affiliations:** 1 The Department of Dermatology, Xiangya Hospital, Central South University, Changsha, Hunan China; 2 National Clinical Research Center for Geriatric Disorders, Xiangya Hospital, Changsha, Hunan China; 3 Hunan Key Laboratory of Skin Cancer and Psoriasis, Xiangya Hospital, Changsha, Hunan China; 4 Hunan Engineering Research Center of Skin Health and Disease, Xiangya Hospital, Changsha, Hunan China; 5 Xiangya Clinical Research Center for Cancer Immunotherapy, Central South University, Changsha, Hunan China

**Keywords:** Cancer therapy, Tumour immunology

## Abstract

Currently, pyroptosis has received more and more attention because of its association with innate immunity and disease. The research scope of pyroptosis has expanded with the discovery of the gasdermin family. A great deal of evidence shows that pyroptosis can affect the development of tumors. The relationship between pyroptosis and tumors is diverse in different tissues and genetic backgrounds. In this review, we provide basic knowledge of pyroptosis, explain the relationship between pyroptosis and tumors, and focus on the significance of pyroptosis in tumor treatment. In addition, we further summarize the possibility of pyroptosis as a potential tumor treatment strategy and describe the side effects of radiotherapy and chemotherapy caused by pyroptosis. In brief, pyroptosis is a double-edged sword for tumors. The rational use of this dual effect will help us further explore the formation and development of tumors, and provide ideas for patients to develop new drugs based on pyroptosis.

## Introduction

**Fig. 1:**
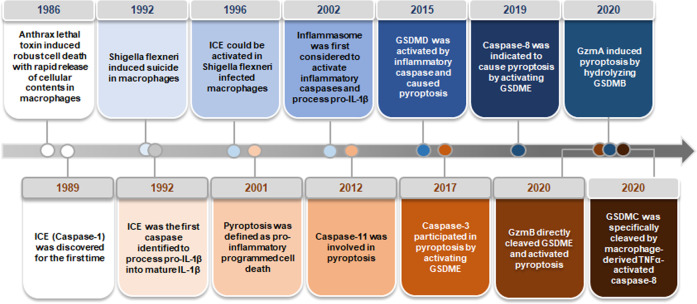
The timeline of pyroptosis

The earliest research on pyroptosis can be traced back to 1986. Friedlander pointed out in the report that treatment of primary mouse macrophages with anthrax lethal toxin (LT) resulted in cell death and rapid release of cell contents.^1^ Cerretti et al. and Thornberry et al. observed that ICE (interleukin-1β-converting enzyme, caspase-1), discovered for the first time in 1989,^2^ was an inflammatory caspase, processing precursor IL-1β into mature IL-1β.^3,4^ In 1992, Zychlinsky et al. discovered pyroptosis for the first time, and they found suicide in the Gram-negative bacterial pathogen Shigella flexneri-infected macrophages.^5^ In 1996, Chen et al. reported that invasion plasmid antigen B (ipaB) of Shigella flexneri could bind directly to ICE and caused the enzyme to be activated in infected macrophages.^6^ This form of cell death was originally deemed to be apoptosis because some of its characteristics were similar to apoptosis, such as caspase-dependent, DNA damage, and nuclear condensation. Afterward, this form of death was observed to be different from apoptosis. In 2001, D’Souza et al. pointed out the term of pyroptosis, which comes from the Greek roots pyro (fire/fever) and ptosis (to-sis, falling), to describe pro-inflammatory programmed cell death.^7^ This is the first definition of pyroptosis, which makes the distinction between pyroptosis and apoptosis (a non-inflammatory program of cell death).^7^ In 2002, Inflammasome was first considered to activate inflammatory caspases and process pro-IL-1β.^8^ Afterward, Petr et al. found non-canonical caspase-11 could induce cell death without relying on caspase-1 when the host was infected with Salmonella.^9^ Pyroptosis was considered to be caspase-1-induced monocyte death for a long time,.^10,11^ Later, it was reported that caspase-1 or caspase-11/4/5 was activated during this process, and gasdermin D (GSDMD) was cleaved, and the N-terminal domain can oligomerize to form pores in the cell membrane, inducing cell membrane rupture.^12^ Interestingly, reports in recent years have shown that GSDMD-mediated pyroptosis can be blocked by some factors. Caspase-3/7 cleaved GSDMD at Asp87 site, causing inactivation of the pyroptotic activity of GSDMD.^13^ In addition, the endosomal sorting complexes required for transport (ESCRT) machinery could eliminate GSDMD pores from the plasma membrane, resulting in the inhibition of GSDMD-mediated pyroptotic cell death and limite the release of IL-1β after the activation of inflammasome.^14^ Humphries et al. found that the intermediate product of tricarboxylic acid cycle, fumarate, also dampened pyroptosis.^15^ Both fumarate and dimethyl fumarate (DMF) could prevent GSDMD from being processed and activated by caspases by succinizing the cysteine of GSDMD.^15^ In 2017, Wang et al. and Rogers et al. showed that chemotherapeutic agents can also induce pyroptosis by activating caspase-3 to shear GSDME.^16,17^ Subsequently, caspase-8 was indicated to cause pyroptosis and affect inflammasome function.^18^ In 2020, it was reported that granzyme B (GzmB) can directly cleave GSDME and activate pyroptosis, further activating the antitumor immune response and inhibiting tumor growth.^19^ In the same year, granzyme A (GzmA) in cytotoxic lymphocytes was found to enter target cells through perforin and induce pyroptosis by hydrolyzing GSDMB at the Lys229/Lys244 site, which renewed our understanding of pyroptosis.^20^ More recently, pyroptosis has made further progress. Hou et al. found that under hypoxia, activated p-Stat3 promoted nuclear PD-L1 translocation. Nuclear PD-L1 and p-Stat3 synergistically promote the expression of GSDMC, and caspase-8 activated by TNF-α derived from macrophages can cleave GSDMC into N-GSDMC at the D365 site, resulting in pyroptosis ultimately^21^ (Fig. 1).

Tumors use multiple strategies to avoid or limit the cell death pathway.^22^ In some cases, tumors can be killed by different ways, such as apoptosis, necrosis, autophagy and pyroptosis.^23,24^ Apoptosis and other modes of death are important anticancer defense mechanisms that have been widely studied, but the relationship between pyroptosis and cancer is not fully understood at present. Pyroptosis is closely related to nervous system diseases, infectious diseases, autoimmune diseases, cardiovascular diseases, and tumors.^25–28^ With further research, the relationship between pyroptosis and tumors is becoming increasingly clear, which provides some strategies for clinical treatments.

We focus on the mechanism, the diverse functions in various tumors and the potential clinical value of pyroptosis. These findings have raised awareness of tumors and identified a number of potential cancer treatments. We look forward to communicating the recent achievements in pyroptosis research and its development in the field of oncology. These findings improve the understanding of tumors and identify a number of potential tumor treatments.

## Gasdermin, the executioner of pyroptosis

Pyroptosis was defined as gasdermin-mediated programmed death in 2015.^29^ The gasdermin superfamily is composed of gasdermin A/B/C/D (GSDMA/B/C/D), gasdermin E (GSDME, also referred to as DFNA5) and DFNB59 (Pejvakin, PJVK) in human (Gsdma1-3, Gsdmc1-4, Gsdmd, Dfna5, and Dfnb59 in mice).^30–34^ Among these conserved proteins, GSDMD and DFNA5 are most deeply studied in pyroptotic death. Except for Pejvakin, all these members consist of two conserved domains, the N-terminal pore-forming domain and the C-terminal repressor domain (PFD and RD).^35–38^ The PFD of most gasdermins could induce pyroptosis, which has not yet been detected for Pejvakin.^17,30,39^ In general, gasdermins maintain oligomerization through the interaction between PFD and RD, and the cytotoxic effects of PFD could be inhibited by RD.^37,38^ When the host is stimulated by a variety of exogenous or endogenous factors, gasdermins are cleaved by some caspases or granzymes, and the N-terminal PFD is dissociated from the C-terminal RD, and then the N-terminal PFD oligomerizes and forms pores in the cell membrane, causing the release of inflammatory molecules and cell pyroptotic death.^30,40,41^ Although a number of gasdermin family proteins have been reported to be linked to human diseases,^20,29,39,42–63^ the specific mechanism and function remain to be studied.

## The characteristics of pyroptosis

Pyroptosis is composed of “pyro” and “ptosis”. “Pyro” means fire, indicating the properties of inflammation of pyroptosis, but “ptosis” means falling, which is consistent with other forms of programmed cell death.^7^ There are some similarities between pyroptosis and apoptosis, such as DNA damage and chromatin condensation.^64,65^ Interestingly, pyroptotic cells emerged swelling and a lot of bubble-like protrusions appear on the surface of the cellular membrane before its rupture.^66^ Similarly, membrane blebbing also occurs during apoptosis, and caspase-3 is necessary for this process.^67–76^ However, the unique morphological characteristics of pyroptosis are obviously different from those of apoptosis. It is generally believed that apoptosis is a safe form of death, but pyroptosis can cause inflammation, activated by extracellular or intracellular stimulation, such as bacterial, viral, toxin, and chemotherapy drugs.^77^ In fact, unlike the explosive rupture associated with necrosis, pyroptosis causes flattening of the cytoplasm due to plasma membrane leakage.^66^ In addition, caspases activation or release of granzymes results in the N-terminal of gasdermin oligomerization and pore formation (1–2 μm in diameter) in the plasma membrane, which allows mature IL-1β/IL-18 with a diameter of 4.5 nm and caspase-1 with a diameter of 7.5 nm to pass through, respectively.^30^ In the meantime, the water entering through the pores causes cell swelling and osmotic lysis, thus resulting in rupture of the plasma membrane and the release of IL-1β and IL-18.^78,79^ Thus, the pyroptotic cells are permeable to 7-aminoactinomycin (7-AAD), propidium iodide (PI), and ethidium bromide (EtBr) because of the low molecular weight of these dyes.^79^ On the contrary, in comparison with pyroptotic cells, apoptotic cells maintain membrane integrity,^80^ so that these dyes can’t stain them.^81–84^ Intriguingly, similar to apoptotic cells, Annexin V also stains pyroptotic cells and the dye binds to phosphatidyl serine (PS).^85^ Therefore, Annexin V cannot differentiate apoptotic cells from pyroptotic cells. In addition, apoptotic bodies are formed in the process of apoptosis, while pyroptotic bodies are formed in the process of pyroptosis.^80^ Interestingly, the diameter of pyroptotic bodies is similar to that of apoptotic bodies, and thier size are both 1–5 µm.^66^

Besides, there is a very special form of DNA damage with dUTP nick-end labeling (TUNEL) staining positive in the early stage of pyroptosis,^79,86–93^ which is different from that of apoptosis. Compared with apoptosis, the intensity of DNA damage is lower in pyroptotic cells. The DNA fragmentation of pyroptosis is random and the nucleus remains intact,^94–97^ while the apoptotic DNA fragment is ordered and the nucleus is fragmented.^23,98–100^ Interestingly, caspase activation is associated with both pyroptosis and apoptosis. Initially, pyroptosis was considered to be caspase-1-related cell death.^5,23,101,102^ It is worth mentioning that recent studies have shown that other caspases, including caspase-3/4/5/6/8/9/11, also cause pyroptosis in other different cells,^16,17,29,39,94,103–105^ and play major roles in innate immunity and tumorigenesis.^106–111^ Caspase-3 was previously thought to be the executor of apoptosis, but it was suggested that caspase-3 can also induce pyroptosis by cleaving GSDME.^16,17,71^ More surprisingly, the apoptosis-related protein caspase-8 can also directly cleave GSDMD to induce pyroptosis.^103^ In addition, the activation of caspase-9 is also involved in pyroptosis by cleaving and activating caspase-3,^112^ and caspase-6 mediates the cleavage of GSDMC.^21^ Although current studies have found that caspase-1 and caspase-4/5/11 are only associated with pyroptosis, while caspase-2, caspase-7, and caspase-10 are only related to apoptosis,^36,113–117^ the connections between caspases and pyroptosis as well as caspases and apoptosis may be reported one after another in the future with the deepening of studies. Apoptosis is affected by the level of ATP, and apoptosis is accompanied by the activation of PARP, causing ATP depletion.^118^ However, the effector proteins of pyroptosis belong to the gasdermin family^23^ (Tables 1 and 2).

**Table 1:** Similarities between pyroptosis and apoptosis

Characteristics	Pyroptosis	Apoptosis
Programmed cell death	Yes	Yes
PS exposure	Yes	Yes
Annexin V staining	Yes	Yes
TUNEL staining	Yes	Yes
DNA damage	Yes	Yes
Chromatin condensation	Yes	Yes
Membrane blebbing	Yes	Yes
The diameters of pyroptotic bodies and apoptotic bodies (1–5 µm)	Yes	Yes
Caspase-3 activation	Yes	Yes
Caspase-6 activation	Yes	Yes
Caspase-8 activation	Yes	Yes
Caspase-9 activation	Yes	Yes

**Table 2:** Differences between pyroptosis and apoptosis

Characteristics	Pyroptosis	Apoptosis
Inflammation	Yes	No
Apoptotic bodies	No	Yes
Pyroptotic bodies	Yes	No
Intact nucleus	Yes	No
Pore formation	Yes	No
Cell swelling	Yes	No
Cell shrink	No	Yes
Osmotic lysis	Yes	No
Membrane integrity	No	Yes
7-AAD staining	Yes	No
PI staining	Yes	No
EtBr staining	Yes	No
Caspase-1 activation	Yes	No
Caspase-4 activation	Yes	No
Caspase-5 activation	Yes	No
Caspase-11 activation	Yes	No
Caspase-2 activation	No	Yes
Caspase-7 activation	No	Yes
Caspase-10 activation	No	Yes
PARP cleavage	No	Yes
Gasdermin cleavage	Yes	No

## Molecular mechanism of pyroptosis

### Canonical pathway

Canonical pyroptotic death is mediated by inflammasome assembly, which is accompanied by GSDMD cleavage and IL-1β and IL-18 release.^10,119–128^ Inflammasomes are multimolecular complexes that are activated when the host is resistant to microbial infection and also facilitate the development of adaptive immune responses. In addition, Inflammasomes are also associated with non-microbial diseases. There is considerable evidence that inflammasomes and their related cytokines play crucial roles in oncogenesis, such as proliferation, metastasis, and invasion.^129–137^ The assembly of inflammasome begins with cytosolic pattern recognition receptors (PRRs, also known as inflammasome sensors), which are capable of recognizing pathogen-associated molecular patterns and danger-associated molecular patterns ((PAMPs and DAMPs).^138,139^ Activation of PRRs promotes downstream signaling pathway and causes type I interferons generation and pro-inflammatory cytokines release.^129,140–144^ PRRs assemble with pro-caspase-1 and ASC to form inflammasomes after stimulation of cells by signal molecules such as bacteria and viruses.^11,141,145–152^

At present, the inflammasome sensors NLRP1, NLRP3, NLRC4, AIM2, and pyrin are able to assemble canonical inflammasomes and have been relatively well studied. Most inflammasomes are constituted by three components: (i) leucine-rich repeat containing proteins (NOD-like receptors, NLRs), (ii) the adapter apoptosis-associated speck-like protein containing a caspase recruitment domain (CARD) (ASC), and (iii) pro-caspase-1. NLRs usually consist of a leucine-rich repeat (LRR), a nucleotide-binding oligomerization domain (NACHT), and a CARD or pyrin domain (PYD),^153–156^ divided into NLRPs or NLRCs according to whether their N terminal contain PYD or CARD. The NLRCs have one or more CARD at their N terminal such as NLRC4, but the NLRPs contain PYD such as NLRP1 and NLRP3.^101,143,157^ Compared with NLRP3, NLRP1 contains extra function-to-find domain (FIIND) and CARD domains at the N terminal.^158^ Human NLRP1 carry three paralogues in mice: NLRP1a/b/c, and these NLRP1 in mice lack PYD.^159^ NLRP1b has been studied relatively extensively and responds to the Toxoplasma gondii, Val-boro-Pro, and the Bacillus anthracis anthrax lethal toxin.^160–163^ NLRP3 senses a variety of stimulus, such as toxins, pathogens, metabolites, crystalline substances, nucleic acids, and ATP.^164^The initial recognition of ligands by various NAIP proteins is necessary for NLRC4 inflammasome activation. The human NAIP can directly bind to the flagellin and proteins of the type 3 secretion system (T3SS).^165–168^ In contrast to human NAIP, the mouse NAIP1, NAIP2, NAIP5, and NAIP6 recognize the needle of T3SS, the inner rod of the T3SS, flagellin, and flagellin, respectively.^165,166,169^ Then, NAIPs induce the recruitment and oligomerization of NLRC4 to form the NLRC4 inflammasome complex, leading to the cleavage of caspase-1 and pyroptosis.^170^ Actually, non-NLRs, such as AIM2 and pyrin can also form inflammasomes. AIM2 consists of an electropositive HIN-200 domain at C terminal and an N-terminal PYD. HIN-200 domain can bind to the electronegative double-stranded DNA (dsDNA),^152,171–177^ and PYD can recruit ASC and pro-caspase-1 to assemble the inflammasome.^152,171–177^ Another inflammasome sensor, pyrin, is activated after the inactivation of small GTPases of the host RHO family. Rho-inactivating toxins, such as the Clostridium difficile glycosyltransferase TcdB, Vibrio parahaemolyticus VopS, and Clostridium botulinum ADP-ribosylating C3 toxin can induce the assembly of pyrin inflammasome. Besides, YopE (the Yersinia pestis GTPase-activating protein) and YopT (cysteine protease) also trigger the activation of pyrin inflammasome.^178–180^ Specifically, mutations of serine residues, such as S208A and S242R, relieve the phosphorylation, resulting in pyrin activation^181–183^ (Fig. 3).

**Fig. 2:**
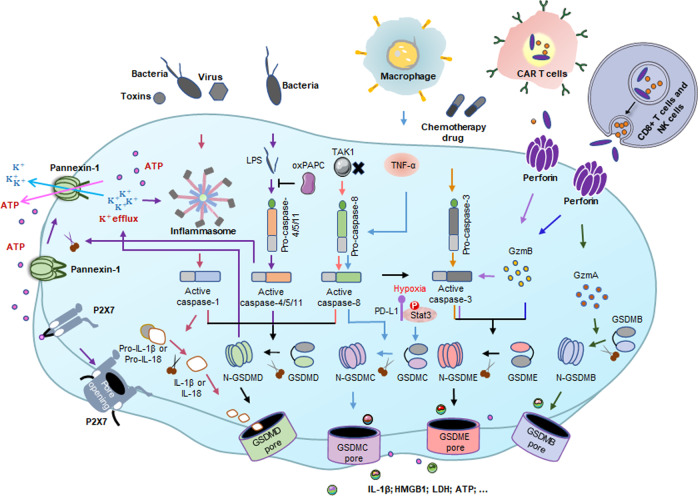
Molecular mechanism of pyroptosis. In the canonical pathway, PAMPs and DAMPs receive intracellular signaling molecule stimulation and assemble with pro-caspase-1 and ASC to form inflammasomes and active caspase-1. Cleaved-caspase-1 cleaves GSDMD and pro-IL-1β/18. N-GSDMD perforates the cell membrane by forming nonselective pores, further causing water influx, lysis, and death. In addition, IL-1β and IL-18 are secreted from the pores formed by N-GSDMD. In the noncanonical pathway, cytosolic LPS activates caspase-4/5 and caspase-11, triggering pyroptosis by cleaving GSDMD. However, oxPAPC competes with LPS to bind caspase-4/1, thus inhibiting pyroptosis. In addition, the cleavage of GSDMD results in efflux of K^+^, ultimately mediating the assembly of NLRP3 inflammasome, resulting in the cleavage of pro-IL-1β and pro-IL-18. The activated caspase-11 also cleaves Pannexin-1, inducing ATP release and P2X7R-related pyroptotic cell death. In the caspase-3-mediated pathway, active caspase-3 cleaves GSDME to form N-GSDME, inducing pyroptosis. In the caspase-8-mediated pathway, inhibiting TAK1 induces the activation of caspase-8, which cleaves GSDMD, resulting in pyroptosis. In addition, under hypoxia conditions, PD-L1 is transferred to the nucleus and regulates the transcription of GSDMC together with p-Stat3, resulting in the conversion of apoptosis to pyroptosis after TNFα-activated caspase-8. In the granzyme-mediated pathway, CAR T cells rapidly activate caspase-3 in target cells by releasing GzmB, and then GSDME- was activated, causing extensive pyroptosis. In adittion, GzmA and GzmB in cytotoxic lymphocytes enter target cells through perforin and induce pyroptosis. GzmA hydrolyzes GSDMB, and GzmB directly activates GSDME

**Fig. 3:**
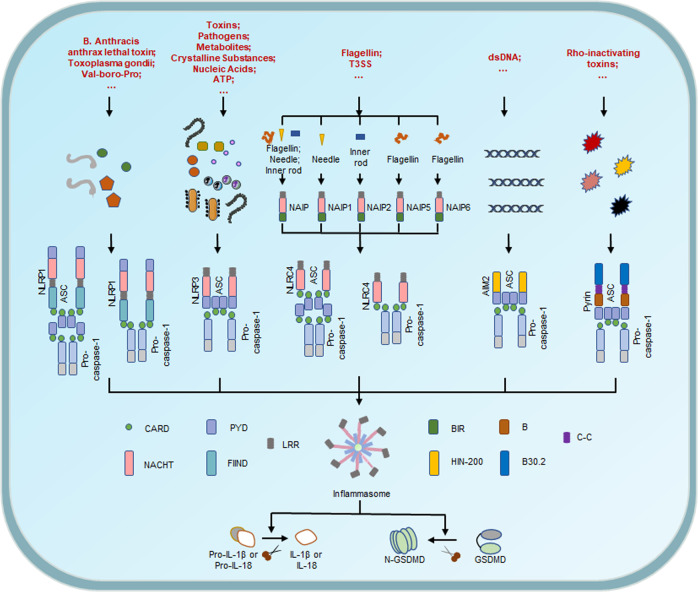
Multiple assembly mechanisms of canonical inflammasomes. The assembly of canonical inflammasomes occurs in response to PAMPs and DAMPs. Inflammasome sensors have interaction with different target ligand. Bacillus anthracis toxin activates the NLRP1 inflammasome, and NLRP1 recruits pro-caspase-1 into the complex via ASC or direct contact with CARD-CARD interactions. A variety of PAMPs and DAMPs activate NLRP3 inflammasome, which is followed by the recruitment of ASC and pro-caspase-1. Human NAIP senses both bacterial flagellin and proteins of T3SS components. Mouse NAIP1, NAIP2 and NAIP5/6 sense needle, inner rod, and flagellin respectively to assemble and activate the NLRC4 inflammasome. NLRC4 activates caspase-1 in an ASC-dependent or ASC-independent manner. AIM2 inflammasome is assembled when AIM2 senses host- or pathogen-derived dsDNA. The pyrin inflammasome is activated by Rho-modifying proteins. Both AIM2 and pyrin activate caspase-1 in an ASC-dependent manner. Activated caspase-1 can not only cleave GSDMD to form N-GSDMD and induce pyroptosis, but also process the precursors of IL-1β/IL-18 to mature IL-1β/IL-18, which are released through the pores formed by N-GSDMD

ASC contains a pyrin domain (PYD) and a caspase activation and recruitment domain (CARD). The CARD is necessary to recruit pro-caspase-1 to form the inflammasome. Some PRRs contain CARD, allowing pro-caspase-1 to also be directly recruited.^101^ After inflammasome assembly, caspase-1 is activated, and is hydrolyzed into 2 fragments, forming a dimer to become mature cleaved caspase-1.^184^ On the one hand, caspase-1 cleaves the caustic executor protein GSDMD at the Asp275 site to form the 22 kDa C-terminus (C-GSDMD) and 31 kDa N-terminus (N-GSDMD). N-GSDMD perforates the cell membrane to form nonselective pores with inner diameters of ~10–14 nm, leading to cell swelling and pyroptosis.^66,185^ On the other hand, caspase-1 also cleaves the precursors of IL-1β and IL-18 to be the mature IL-1β and IL-18, which are released through the pores formed by GSDMD, resulting in pyroptosis.^3,4,11,29,31,40^ Notably, after inflammasome activation, cells may undergo pyroptosis or release cytokines without cell death, but the specific mechanism is unknown. Some studies have suggested that the Toll-like receptor adapter protein SARM is involved in this regulation^186^ (Figs. 2 and 3).

### Non-canonical pathway

In the non-canonical pyroptosis pathway, the upstream sensory complexes of human caspase-4/5 (mouse orthologs caspase-11) are absent, and these caspases can be activated by directly binding to intracellular lipopolysaccharide (LPS) through the N-terminal CARD.^187^ It is worth noting that the oxidized phospholipid 1-palmitoyl-2-arachidonoyl-sn-glycero-3-phosphorylcholine (oxPAPC, a TLR4 agonist) is in competition with LPS to bind caspase-4/11, and reduces the non-canonical inflammasome in macrophages, but not in dendritic cells.^188^ Activated caspase-4/5/11 can also cleave GSDMD into N-GSDMD, which is oligomerized and transferred to the cell membrane to form plasma membrane pores ultimately.^189^ However, caspase-4/5/11 cannot cleave pro-IL-1β/pro-IL-18, but they are able to mediate the maturation and secretion of IL-1β/ IL-18 through the NLRP3/caspase-1 pathway in some cells.^23^ In addition, GSDMD is cleaved by caspase-4/5/11, resulting in the efflux of K^+^,^29^ inducing the assembly of NLRP3 inflammasome, eventually leading to pyroptosis.^190–192^ Of note, Yang et al. found that Pannexin-1 is another key protein that mediates pyroptotic cell death in the non-classical pathway induced by caspase-11.^193^ Under the stimulation of LPS, activated caspase-11 can specifically shear and modify Pannexin-1, causing the release of cellular ATP, thereby inducing pyroptosis mediated by ion channel P2X7 receptor.^193^ Importantly, murine BMDMs with the deficiency of Pannexin-1 could also cause K^+^ efflux and NLRP3 inflammasome-mediated caspase-1 cleavage with P2X7 independent,^193^ which coincides with a published report in 2011.^115^ Additionally, knockout Pannexin-1 in mice protects against endotoxin shock, suggesting that selective K^+^ channels modulate non-canonical NLRP3 activation.^193^ (Fig. 2).

### Caspase-3/8-mediated pathway

The members of gasdermin protein family are highly conserved in structure. Except for DFNB59, all gasdermins contain C-terminal and N-terminal domains, and the N-terminus is the pyroptosis executor.^30^ Previously, apoptosis-related caspases (such as caspase-3/8) were thought to be unable to stimulate gasdermin to induce pyroptosis, while it was shown that chemotherapeutic drugs could induce caspase-3-mediated GSDME cleavage in with high GSDME expression and form N-GSDME termini, which caused pyroptosis of tumor cells.^16,17^ In addition, it has been found in mouse macrophages that the effector protein YopJ expressed during Yersinia infection can inhibit TGF-β-activated kinase 1 (TAK1) and induce caspase-8-related cleavage of GSDMD,^103,104^ which further improves and expands the understanding of pyroptosis. Intriguingly, PD-L1 converts TNF-mediated apoptosis into pyroptosis in breast cancer cells. Under hypoxia conditions, p-Stat3 promotes nuclear translocation of PD-L1, and they jointly enhance the transcription of GSDMC. Under the stimulation of TNF-α, Caspase-8 specifically lyses GSDMC to produce N-GSDMC, and forms pores on the cell membrane to induce pyroptosis. Macrophage-derived TNF-α-induced tumor pyroptosis requires nuclear PD-L1, caspase-8, and GSDMC In vivo. In addition, Antibiotic chemotherapy drugs can also trigger caspase-8/GSDMC-mediated pyroptotic death in breast cancer cells^21^ (Fig. 2).

### Granzyme-mediated pathway

In 2020, Liu et al. reported that CAR T cells rapidly activated caspase-3 in target cells by releasing GzmB, and then the caspase-3/GSDME-mediated pyroptotic pathway was activated, causing extensive pyroptosis.^194^ More recently, researchers found that GzmB directly cleaved GSDME and induced pyroptosis, further activating the antitumor immune response and inhibiting tumor growth.^19^ Subsequently, it was reported that natural killer cells and cytotoxic T lymphocytes (CTLs) killed GSDMB-positive cells by pyroptosis. The killing effect resulted from GSDMB cleavage at the Lys229/Lys244 site by lymphocyte-derived GzmA. GSDMB is highly expressed in some tissues, especially in the epithelium of the digestive tract, including derived tumors. This study showed for the first time that gasdermin could be hydrolyzed by GzmA at non-aspartic acid sites and form pores, redefining the idea that pyroptosis can only be activated by caspases^20^ (Fig. 2).

## Role of pyroptosis in tumors

Pyroptosis may act as a pivotal part in multiple tumors. With further research, the relationship between pyroptosis and tumors has become increasingly understood and provides some inspiration for clinical treatments.

### Pyroptosis and melanoma

Melanoma is a common malignant tumor and is typically associated with BRAF and NRAS mutations.^195–199^ Previous work by Corey et al. found that the pyroptosis-related protein GSDME from B16-Ova cell lines exerted a tumor suppressive effect.^35^ In addition, it has been found that melanoma cells with GSDME deficiency generate larger tumors than wild-type melanoma cells; thus, GSDME may have real tumor inhibitory activity.^35^ In 2020, the combination of BRAF and MEK inhibitors was reported to induce GSDME-dependent pyroptosis in melanoma cells.^200,201^ In immunocompetent mice, BRAF inhibitor + MEK inhibitor caused an increase in CD4+ T cell and CD8+ T cell infiltration and a decrease in myeloid-derived suppressor cells (MDSCs) and tumor-associated macrophages (TAMs).^200^ In BRAF inhibitor + MEK inhibitor-resistant tumors, the loss of pyroptosis and GSDME cleavage induced by BRAF inhibitor + MEK inhibitor was associated with a decreased antitumor immune response.^201^ DHP1808, a new Hsp90/PI3K inhibitor, can effectively induce apoptosis of melanoma cells by interfering with the interaction between Hsp90 and EGFR and inhibiting the downstream MAPK signaling pathway. However, a recent study found that DHP1808 induced less pyroptosis than the combination of Hsp90 and PI3K inhibitors in tumor and intestinal tissue, indicating that DHP1808 is a safe drug and worthy of further development.^202^ Moreover, z-YVAD-FMK inhibited the ability of the HSV-2 mutant lacking R1 protein kinase activity (ΔPK) to induce melanoma cell death, showing that ΔPK could induce pyroptosis.^203^ Interestingly, increased etoposide resistance in melanoma cells led to decreased GSDME mRNA levels due to increased cell sensitivity to the activation of caspase-3-dependent signaling pathways, triggering programmed cell death and suggesting that GSDME may be involved in the multimodal mechanism that led to drug resistance in the treatment of malignant melanoma.^204^ Iron was involved in redox cycle and the activation of ROS,^205,206^ and ROS was thought to be related to cancer.^207,208^ Generally, Zhou et al. found that carbonyl cyanide m-chlorophenyl hydrazone (CCCP) activated oxidative stress, and iron significantly enhanced this effect, followed by Tom20 oxidation and oligomerization in melanoma A375 cells.^112^ Tom20 promoted the recruitment of Bax to mitochondria, then cytochrome c was released into the cytoplasm and caspase-3 was activated, which eventually induced the cleavage of GSDME and caused pyroptosis,^112^ indicating that iron can enhance the sensitivity of drugs that activate ROS in vivo, and amplify ROS signals to drive pyroptosis. It also reveals a potential iron-based intervention strategy for treatment of melanoma patients.^112^ In addition, eEF-2K regulated the pathway linking autophagy and pyroptosis.^209^ Inhibition of eEF-2K makes melanoma cells more sensitive to doxorubicin.^209^ However, the specific mechanism of eEF-2K regulating GSDME remains to be studied.^209^ It has been reported that melanoma cells overexpressing GSDMB show obvious characteristics of pyroptosis. The mechanism of GSDMB-induced pyroptosis is not related to the classic caspase pathway but to the protein cleavage mediated by perforin and granzyme, which are produced by cytotoxic particles.^20^ It was reported that the polymorphism of NLRP1/NLRP3 can affect the susceptibility of malignant melanoma (MM) and the occurrence of nodular melanoma (NM).^210^ Unlike other NLRPs, the C-terminal domain of NLRP1 contains a CARD-binding motif that interacts with pro-caspase-1 through CARD-CARD.^114,211,212^ Thus, ASC does not participate in the NLRP1 activation of caspase-1.^114,211,212^ In 2017, it was found that NLRP1 could not only synergistically promote caspase-1-mediated inflammasome activation, but also inhibit caspase-2/9 related mitochondrial apoptosis of melanoma, thus promoting the tumorigenesis of human melanoma.^213^ Therefore, NLRP1 may be a new target for the treatment of human melanoma. Additionally, The inhibition of B16F10 lung metastasis in wild type mice was mediated by NK cells,^214–217^ and NLRP3 played an important role in this process.^218^ After intravenous injection of B16F10 melanoma cells, wild-type mice had significantly more lung metastases than NLRP3 knockout mice.^218^ In addition, the protective effect of NLRP3 deletion on lung metastasis of B16F10 depended on NK cells, but not on CD4+ and CD8+ T cells.^218^ The absence of NLRP3 increased the number of activated NK cells, secreted more IFN-γ, and killed more tumor cells to reduce B16F10 lung metastasis.^218^ This may seem strange, since the loss of host NLRP3 inhibited the release of pro-inflammatory cytokines, which contributed to NK cell activation, but the tumor microenvironment was unique. Intervention of NLRP3-dependent immunosuppressive pathway may be a potential therapeutic strategy to enhance host resistance to melanoma metastasis.^218^ In 2020, Okamoto et al. found that human melanoma cells spontaneously secreted active IL-1β through the structural activation of NLRP3 inflammasome, thus showing the characteristics of autologous inflammatory diseases.^219^ Interestingly, NLRP3 can also destroy the effect of anti-tumor vaccine. The survival rate of mice with NLRP3 deficient bearing melanoma cell line B16F10 based on dendritic cell vaccination was significantly higher than that of their respective control groups. The increase in survival was attributed to the lower number of MDSCs in NLRP3-deleted tumors, suggesting that NLRP3 played a role in promoting MDSCs-to-TME migration.^220^ Surprisingly, NLRC4 seemed to play a role different from NLRP1 and NLRP3 in melanoma, which amplified inflammatory signals in macrophages and inhibited the growth of melanoma tumors independent of inflammasome assembly.^221^ NLRC4 inflammasome consists of NLRC4, ASC, and caspase-1.^222,223^ Compared with wild-type mice, the tumor volume of NLRC4 gene deficient mice was significantly larger, while the tumor size of mice with knockout inflammasome components ASC and caspase-1 had no significant change, suggesting that NLRC4-mediated regulation of antitumor growth is not associated with NLRC4 inflammasome activation in B16F10 melanoma model.^221^ In addition, the researchers found that the loss of NLRC4 was related to the inability of CD4+ and CD8+ T cells to produce INF-γ. Although immunotherapy for melanoma that enhances endogenous effector T cell response has yielded promising clinical results,^224–228^ further studies are needed to determine specifically whether the reduction of chemokine production and the population of CD4+ and CD8+ T cells producing INF-γ directly affect tumor growth. Inflammasome regulates the activation of caspase-1 by ASC, which contains a CARD. However, ASC plays multiple physiological roles in the pathogenesis of melanoma through different regulation of NF-κB and IL-1β.^212^ On the one hand, ASC appeared to induce metastatic melanoma by activating caspase-1-dependent IL-1β secretion and enhancing its autonomous inflammatory NF-κB activity, suggesting that ASC is a tumor-promoting factor in metastatic melanoma; On the other hand, ASC was highly expressed in primary melanoma, which reduced the phosphorylation of IKKα/β and inhibited the activity of NF-κB, thus playing an anti-tumor role.^212^ These studies deeply emphasized the cell type and tissue-specific role of inflammasome components in melanoma. In-depth study of ASC and its upstream and downstream molecules or pathways may deepen our comprehension of the molecular mechanism of melanoma and provide new molecular targets for the design of anti-melanoma drugs.^212^ In particular, pleiotropic cytokine IL-1β/IL-18 also promote the growth, angiogenesis and metastasis of melanoma in an autocrine or paracrine manner.^229–231^ IL-1β-activated hepatic sinusoidal endothelium (HSE) cells release very late antigen-4 stimulating factor to enhance the adhesion of B16 melanoma (B16M) cells to HSE.^232^ Besides, IL-1β and IL-18 are involved in the process of hepatic metastases of B16M in vivo. The soluble products of B16M cells caused HSE to release TNF-α, IL-1β, and IL-18.^233^ IL-18 further increased the expression of adhesion molecule-1 (VCAM-1) and promoted the adhesion of melanoma cells.^233^ PD-1, expressed by activated mature NK cells in lymphoid organs of tumor carriers, can be also upregulated by IL-18.^234^ Inhibition of IL-18 or IL-18 binding proteins in melanoma was sufficient to stimulate NK cell-dependent immune surveillance in melanoma models,^235^ indicating that IL-18 can be used as an immunosuppressive cytokine in melanoma, and anti-PD-1 antibody has a new clinical application in human malignant tumors that produce IL-18.

### Pyroptosis and breast cancer

Breast cancer is one of the most frequent malignancies.^236–241^ A high level of GSDMB in breast cancers was related to tumor progression, and overexpression of GSDMB indicated a poor response to targeted treatment of HER-2.^53^ This means that GSDMB could be a novel prognostic marker for tumors. In addition, high levels of GSDMC were associated with poor survival in breast cancer.^21^ A variety of antibiotics such as doxorubicin, daunorubicin, actinomycin D, and epirubicin could promote the expression of nuclear PD-L1 and GSDMC, and facilitate the activation of caspase-8, resulting in pyroptotic death in breast cancer cells.^21^ Low expression of GSDME was found in many cancers, and low levels of GSDME were also associated with low survival of breast cancer patients.^242^ In 2018, it was reported that docosahexaenoic acid exerted anti-tumor effects and induced pyroptosis in breast cancer cells, but this process could be suppressed by caspase-1 inhibitors.^243^ In addition, p53 induced the expression of GSDME through a specific p53 binding site in GSDME.^244^ Interestingly, the DAC restored the induction of GSDME by p53 and two other p53 family genes (p63gamma and p73beta), indicating that GSDME may be a transcriptional target of the p53 family.^245^ Furthermore, the GSDME mRNA level in breast cancer tissues was downregulated because the GSDME promoter was methylated more frequently in tumor tissues than in normal tissues. In addition, GSDME enhanced resistance to triple-negative breast cancer by activating pyroptosis. GSDME methylation was correlated with lymph node metastasis in breast cancer patients, suggesting GSDME promoter methylation is a novel molecular biomarker for human breast cancer.^246^ P2X7 signaling pathway has been found to be related to tumorigenesis.^247–251^ Interestingly, a study published in 2015 showed that Ivermectin (an antiparasitic agent approved by FDA)-induced breast cancer pyroptosis depended on the release of ATP and downstream signals of P2X7 receptors, which can be reversed by inhibiting ROS, Ca^2+^/Calmodulin-dependent protein kinase II (CaMKII) or blocking mitochondrial permeability transition pore (MPTP). Ivermectin induced autophagy and release of ATP and HMGB1.^252^ Autophagy can not only resist many chemotherapeutic drugs, but also enhance immunogenicity and make cancer cells more sensitive to immune-mediated killing.^253,254^ The P2X4/P2X7/Pannexin-1 cascade regulated autophagy and ATP release, and participated in the recruitment and activation of many kinds of immune cells, which acted as a positive feedback loop.^255,256^ Therefore, the anticancer properties of Ivermectin are not only reflected in its direct cytotoxicity. The enhanced P2X4/P2X7 signal can be further associated with the tumor microenvironment rich in ATP, providing clues for purinergic receptors to regulate tumor selectivity and affect the potential of tumor immunotherapy.^252^ The pyroptosis-related inflammasome and cytokine IL-1β also appeared to be significantly associated with the high recurrence rate of breast cancer, and were very important for the proliferation, angiogenesis, migration, and invasion of breast cancer.^229^ Compared with the wild type mice, mice with inflammasome deficiency showed higher survival rate.^257^ After injected orthotopically with PyT8 tumor cells, the tumor growth and metastasis abilities of NLRP3 knockout mice were also lower than that of wild type mice.^257^ Chemokine (C–C motif) ligand 2 (CCL2), a member of the C–C chemokine family, recruited myeloid cells to inflammatory sites and was shown to promote macrophage infiltration into tumor tissue.^258–260^ Guo et al. found that IL-1β stimulated CCL2 expression in macrophages and tumor cells.^257^ Importantly, targeting inflammasome/IL-1 signaling pathway inhibited the growth and metastasis of breast cancer in vivo.^257^ These results suggest that the inhibition of inflammasome/IL-1 signaling pathway in tumor microenvironment may provide a new perspective for the treatment of breast cancer. Pyroptosis and IL-1β produced by pyroptosis were detected in periodontal inflammation (PI) patients, as well as in mouse models.^261^ PI and IL-1β motivated the expression of CCL5, CXCL12, CCL2, and CXCL5, which recruit MDSC and macrophages to promote breast cancer metastasis.^261^ Similarly, the use of anti-CCL5 antibodies to target the CCL5 (a prominent marker of poor prognosis in inflammatory breast cancer detected in many clinical specimens of breast cancer^262–266^) was described as reducing the immunosuppressive activity of MDSCs and decreasing breast cancer metastasis.^267^ There was a direct relationship between chronic PI and the risk of breast cancer metastasis, and PI induced the recruitment of macrophages, cancer cells, and MDSC.^267^ As a result, inflammasome inhibitors are suggested to suppress tumor progression.^267^ Intervention of PI may be an effective way to prevent breast cancer from metastatic to the head and neck. Obesity was related to the elevated risk of ER-positive breast cancer.^268–270^ Generally, tumor microenvironment in obese patients recruited tumor infiltrating myeloid cells with the activation of NLRC4 inflammasome, and causes the production of IL-1β.^271^ IL-1β promoted the progression of breast cancer through adipocyte-originated angiogenesis and the expression of vascular endothelial growth factor A (VEGFA).^271^ In 2020, Jiao et al. found NLRP1 inflammasome was involved in breast cancer cell pyroptosis triggged by human umbilical cord MSCs (hUCMSCs)-CM. Surprisedly, in caspase-4-deficient MCF7 cells, hUCMSC-CM mainly induced pyroptosis through classical pathway, while in NLRP1-deficient MCF7 cells, pyroptosis was mainly induced by non-classical pathway.^272^ These studies facilitate the identification of potential breast cancer treatment targets.

### Pyroptosis and colorectal cancer

Colorectal cancer is a heterogeneous disease associated with genetic mutations, age, family history, ethnicity, and lifestyle.^273–278^ The downregulation of GSDMC led to significant decreases in the proliferation and of colorectal cancer cells, while GSDMC overexpression promoted cell proliferation, tumorigenesis suggesting that GSDMC may be a promising therapeutic target in colorectal cancer.^54^ GSDMD was downregulated in carcinoma cells, while it was slightly overexpressed in normal colorectal epithelial cells.^279^ In 2019, Ibrahim et al. found that colorectal cancer and normal tissue could be distinguished accurately by a combination of two CpGs regardless of age or stage, indicating that GSDME is a novel diagnostic index for colorectal cancer.^280^ Lobaplatin-induced ROS/JNK signal transduction, which induced GSDME-mediated pyroptosis in colon cancer cells.^281^ Interestingly, the lncRNA RP1-85F18.6 is overexpressed in colorectal cancer, playing a key role in tumorigenesis and inhibiting pyroptosis in colorectal cancer cells.^279^ Pyroptosis-associated inflammasomes have been shown to inhibit tumorigenesis. It was reported that Nlrp1b (−/−), Nlrp3 (−/−), Nlrc4 (−/−), Aim2 (−/−), and pyrin (−/−) mice showed significant increases in inflammation, morbidity, and tumorigenesis compared with those of wild-type animals.^282–289^ Besides, compared with that of normal tissues, the expression of NALP1 was decreased in colon cancer tissue.^290^ In addition, DAC could restore the expression of NALP1, thus inhibiting the occurrence of colon cancer, indicating that NALP1 is a potential therapeutic marker for colorectal carcinoma.^282,290^ Dextran sodium sulfate (DSS) destroys the epithelial barrier, leading to a large amount of inflammation caused by intestinal microflora. Azoxymethane (AOM) is a powerful carcinogen, which causes DNA damage to epithelial cells. Multiple cycles administration of DSS promotes chronic inflammation, thereby accelerating the development of colorectal cancer induced by AOM. Although DSS-treated mice with loss of inflammation are generally more likely to develop induced colitis than wild-type mice, the results may be different on specific NLRP. Some studies have also found that NLRP1b, NLRP3, and pyrin protected against colorectal cancer by facilitating the secretion of IL-18 to promote epithelial barrier regeneration during the early stages of colorectal cancer.^283,284,291–294^ Although DSS-treated mice with loss of inflammasome are generally more likely to develop colitis than wild-type mice, the results may be different on specific NLRPs. It has been shown that in the DSS and AOM + DSS model, the inflammasome components possessed protective effects in acute and recurrent colitis and colitis-associated cancer (CAC). Mice with ASC and caspase-1 deficiency showed increased morbidity. The elevated tumor load was associated with decreased IL-1β and IL-18 levels. In addition, acute colitis, recurrent colitis, and CAC were increased in Nlrp3 knockout mice, but disease progression in NLRC4-deletion mice was not significantly different from that in wild-type mice.^283^ Lack of Nlrp3 inflammasome can also lead to impaired IL-18 signaling on NK cells and aggravate the metastatic growth of colorectal cancer growth in the liver.^295^ Interestingly, immune surveillance caused by inflammasome was dependent on NK cells but did not require T and B cells.^295^ Besides, mice with Nlrp3 or caspase-1 deficiency were highly sensitive to inflammation induced by AOM / DSS, and the burden of colon tumor was significantly elevated. This was a consequence of markedly reduced IL-18 levels in mice lacking components of the Nlrp3 inflammasome.^284^ What’s more, defective inflammasome activation resulted in loss of epithelial integrity, leading to numerous leukocyte infiltration, and an increase in chemokines in the colon. This is due to the reduction of IL-18 in mice, causing higher mortality. Therefore, the Nlrp3 inflammasome plays a vital role in maintaining intestinal homeostasis and preventing colitis.^291^ These data suggest that the inflammasome can reduce colitis and CAC. However, another report showed that in comparison to wild-type mice, the severity of colitis in NLRP3-deficient mice was less severe after oral administration of DSS.^296^ It is possible that different experimental methods and inherent differences in variables have caused significant differences between these studies. Interestingly, a report showed that mice with inflammasome component deficiency, such as NLRP3−/− mice, may be characterized by changes in intestinal microflora.^297^ This suggests that alteration in intestinal flora between different animal facilities may also be responsible for the differences observed in these studies. In addition, mice lacking ASC and caspase-1 are also prone to DSS-induced colitis and colon-related colorectal cancer,^283,284,291,294,297^ which provides a great deal of evidence for the protective role of inflammasome in colorectal cancer inflammatory models. In addition, IL-18 is also closely related to the development of colorectal cancer. On the one hand, injecting recombinant IL-18 into caspase-1−/− mice reduced disease progression or outcome in response to dextran sulfate sodium DSS and AOM.^283^ On the other hand, deletion of IL-18 or its receptor IL-18R in intestinal epithelial cells induced resistance to DSS-induced colitis in mice.^298^ Therefore, local production of IL-18 in intestinal epithelial cells in the early stage could be beneficial to epithelial repair after injury, while excessive production of IL-18 in the late stage during chronic inflammation may promote tumor development, indicating that this pyroptosis-related cytokine could be regarded as a potential candidate for immunotherapy in the treatment of some colorectal cancers.

### Pyroptosis and gastric cancer

Gastric cancer is a malignancy that begins in stomach cells and has poor prognosis and high mortality.^299–305^ A study showed that GSDMA was a tumor suppressor gene in gastric cancer,^306^ but GSDMB was overexpressed and in some gastric cancer cells and could act as an oncogene. GSDMB was highly expressed in most cancerous tissue samples, but not in the majority of normal gastric samples, and may be associated with invasion.^307^ In contrast, GSDMC was downregulated in gastric cancer, indicating that it may function as a tumor-inhibiting factor. Similarly, the expression level of GSDMD was low in gastric cancer cell lines and models.^306,308^ In addition, the downregulation of GSDMD could activate the signal transducer and activator of STAT3 and PI3K/PKB signaling pathways, and regulate cell cycle-related proteins to accelerate S/G2 phase cell transition, suggesting that GSDMD downregulation may be beneficial for the treatment of gastric cancer.^309^ It was found that chemotherapeutic drugs induced pyroptosis rather than apoptosis in gastric cancer cells with high expression of GSDME. After treating the gastric cancer cell lines with 5-fluorouracil (5-FU), the cells appeared to exhibit the characteristic of pyroptosis.^299^ Pyroptosis is mainly caused by microbial infection,^11,310^ and studies have found that persistent infection of Helicobacter pylori contributes to the progression of multiple gastric and extra-gastric diseases.^301,311–315^ Importantly, Semper et al. reported that IL-1β produced by Helicobacter pylori in innate immune cells was mainly related to the activation of the NLRP3 inflammasome,^316^ which was consistent with a study that IL-1β secreted by dendritic cells infected by Helicobacter pylori was associated with the synergism between TLR2/NOD2 and NLRP3 inflammasome.^317^ NLRP3 played a role in the gastric cancer development in an inflammasome pathway-dependent or independent way.^318^ The high expression of NLRP3 in gastric cancer accelerated the activation of NLRP3 inflammasome and the release of IL-1β by macrophages.^318^ Besides, Helicobacter pylori infection significantly promoted the expression of NLRP3, but inhibited the expression of miR-22. miR-22 was an inhibitor of NLRP3, and directly attenuated the carcinogenic activity of NLRP3 in vitro and in vivo to maintain the balance of gastric microenvironment.^318^ However, the mechanism between the NLRP3 inflammasome and gastric carcinogenesis needs to be further elucidated. Gastric chronic inflammation caused by Helicobacter pylori infection and the release of IL-6, IL-1β, TNF-α can affect the proliferation of gastric cell.^319,320^ IL-18 produced thrombospondin-1 (a proangiogenic factor) through JNK pathway, which stimulates angiogenesis of gastric cancer cells expressing IL-18R, and affects carcinogenesis and metastasis of gastric cancer.^321–324^ IL-1β was also related to an increased risk of spontaneous gastric inflammation and gastric cancer.^325^ Overexpression of IL-1β in the stomach contributed to the development of gastric cancer by increasing the number of MDSCs.^325^

### Pyroptosis and hepatocellular carcinoma

Hepatocellular carcinoma is the second leading cause of cancer-related death worldwide, and its incidence is expected to further increase globally.^326–333^ It was reported that DFNA5 was significantly expressed at low levels in hepatocellular carcinoma cells. DFNA5 overexpression resulted in the inhibition of cell proliferation.^334^ Furthermore, berberine inhibited the viability of HepG2 cells through caspase-1-mediated pyroptosis.^335^ Interestingly, caspase-1-mediated pyroptosis may also play a role in APOL1-induced cytotoxicity.^336^ A great deal of evidence has confirmed that NLRP3 inflammasome is associated with liver failure and liver disease.^337,338^ In a previous study, Wei et al. found that the loss of the NLRP3 inflammasome contributed to the progression of hepatocellular carcinoma. In addition, 17β-estradiol (E2) suppressed the tumor progression of hepatocellular carcinoma by activating the NLRP3 inflammasome.^339,340^ In 2019, that group demonstrated that E2-induced activation of the NLRP3 inflammasome resulted in caspase 1-mediated pyroptosis.^341^ Besides, targeting the NLRP3 inflammasome may exert inhibitory effects on proliferation, metastasis, and invasion of hepatocellular carcinoma,^342^ indicating that it is necessary to understand the exact mechanism of inflammasome in the proliferation, metastasis and aggression of hepatocellular carcinoma. In addition, Wei et al. found that autophagy inhibition enhanced pyroptosis in hepatocellular carcinoma cells,^341^ suggesting that pyroptosis may be a potential treatment strategy for hepatocellular carcinoma. Hepatic steatosis or cirrhosis is closely related to the occurrence of hepatocellular carcinoma.^343–346^ New evidence suggests that inflammasome plays an important role in non-alcoholic fatty liver disease, which is a series of metabolic disorders ranging from steatosis (NAFL) to steatohepatitis (NASH) to liver cirrhosis. Moreover, it has also been reported that inflammasome activity can inhibit hepatic steatosis.^347–349^ The activation of inflammasome and the production of IL-1β induced by endoplasmic reticulum stress produced a positive feedback loop, which amplified the inflammatory response, resulting in hepatic steatosis and injury.^347^ Similarly, AIM2 was low in hepatocellular carcinoma, the loss of which was significantly associated with mTOR-S6K1 pathway activation and advanced hepatocellular carcinoma progression.^350^ However, the role of inflammasome in hepatocellular carcinoma has not been deeply explored, and its role in the occurrence and development of hepatocellular carcinoma needs further clinical and experimental research.

### Pyroptosis and lung cancer

Lung cancer is one of the primary causes of death and the most common cancer in the world.^351–358^ GSDMD was found to be upregulated in non-small cell lung cancer (NSCLC).^359^ In addition, a high level of GSDMD promoted tumor metastasis and suggested a poor prognosis in LUAD.^359^ Interestingly, in GSDMD‑deficient tumor cells, activation of the pyroptotic signaling pathway (NLRP3/caspase‑1) induced apoptosis but not pyroptosis.^359^ Moreover, silencing GSDMD inhibited tumor proliferation by attenuating the EGFR/Akt signaling pathway in NSCLC.^359^ In 2019, Xi et al. demonstrated that GSDMD colocalized with GzmB near immune synapses, and the defect in GSDMD alleviated the cytolytic ability of CD8+ T cells, suggesting that GSDMD is essential in the immune response of tumor cells.^360^ GSDME is widely expressed in different molecular subtypes of lung cancer. GSDME or caspase-3 depletion significantly weakened GSDME-dependent pyroptosis in A549, PC9 or NCI-H3122 cells.^361^ Zhang et al. showed that both paclitaxel and cisplatin could obviously induce apoptosis in A549 cells, but some of the dying cells showed a morphology that was highly characteristic of pyroptosis.^362^ In A549 cells, cisplatin triggered more severe pyroptosis than paclitaxel, indicating that cisplatin may have an additional advantage over paclitaxel in lung cancer with high expression of GSDME.^362^ Polyphyllin VI (PPVI) induced caspase-1-mediated pyroptosis in NSCLC through the ROS/NF-κB/NLRP3/GSDMD signaling axis, which suggests that PPVI is a potential new therapeutic agent for NSCLC treatment.^363^ Both AIM2 and NLRP3 inflammasome were highly expressed in NSCLC.^364,365^ NSCLC cells with high metastatic expressed relatively more inflammasome components and released more interleukin than cells with low metastatic; Besides, cisplatin-sensitive NSCLC cells also possessed higher levels of inflammasome components interleukin release than cisplatin-resistant NSCLC cells; in addition, in resected lung cancer tissues, upregulation of inflammasome components and interleukin release also observed in high-grade adenocarcinoma (ADC) than low-grade ADC, suggesting that inflammasomes may be conducive to chemotherapy sensitivity of lung cancer.^364^ Taken together, these findings show that the levels of inflammasomes in various lung tumors are dependent on the subtypes, tumor stages, invasive potential, and chemosensitivity of lung cancer, indicating that inflammasomes may be important biomarkers and promising regulators of lung cancer.^364^ Additional studies have also demonstrated the relationship between inflammasome and lung cancer. For instance, the researchers found that inhibition of NF-κB, a transcription factor associated with the initiation of inflammasome,^366–368^ led to a reduction in the viability and proliferation of lung cancer cells.^369,370^ LncRNA-XIST was an oncogene in multiple tumors and played a role the regulation of NSCLC cell proliferation, invasion, and metastasis,^371–382^ which significantly highly expressed in NSCLC tissues and cell lines compared with normal tissue or cells,^383^ regulating the expression level of SOD2 (a mitochondrial antioxidative enzyme can be inhibited by miR-335 in renal senescence^384^) by targeting miR-335 (a negative tumor regulator that inhibits the progression of NSCLC^385–387^). Interestingly, knocking down LncRNA-XIST increased ROS level and activated NLRP3 inflammasome in A549 cells,^383^ and the generation of ROS was reported to induce pyroptosis.^388–390^ Besides, ROS scavenger N-acetyl cysteine (NAC) can block pyroptosis in NSCLC cell induced by the knock down of lncRNA-XIST, and the inhibition of cell proliferation can also be reversed by pyroptosis inhibitor necrosulfonamide (NSA).^383^ Generally, downregulation of lncRNA-XIST inhibits the development of NSCLC by activating miR-335/SOD2/ROS cascade-related pyroptosis.^383^

### Pyroptosis and cervical cancer

Cervical cancer is one of the most common female malignant tumors in worldwide and is one of the primary causes of tumor death among women.^391–396^ The researchers found that HeLa cells with GSDMB overexpression showed obvious characteristics of pyroptosis.^20^ In addition, the release of GzmB by immune cells cleaved GSDME and promoted HeLa cell pyroptosis, which is an important finding for understanding the interaction between GSDME-mediated cell death and the immune system.^19^ The results indicate that there is a positive feedback relationship between pyroptosis and the immune response. Indeed, normal cervical epithelial cells released less IL-18 and IL-1β than cervical cancer cells.^397^ Sirtuin 1 (SIRT1) was overexpressed in HPV-infected cervical cancer cells, and knocking down SIRT1 caused cervical cancer cells to undergo pyroptosis, as well as highly express AIM2 and its downstream genes associated with the inflammasome response. Thus, SIRT1 may be a potential therapeutic target for cervical cancer.^398^ More recently, Song et al. discovered that human papillomavirus E7 (HPV E7, a sexually transmitted DNA virus) could suppress pyroptotic cell death induced by dsDNA transfection. HPV E7 recruited E3 ligase TRIM21 to cause ubiquitination and degradation of IFI16 inflammasome, resulting in suppression of pyroptosis and self-escape of immune monitoring, revealing a significant immune escape mechanism in HPV infection, which may provide a novel target for the development of immunotherapy strategy for effective restoration of antiviral immunity.^399^

### Pyroptosis and leukemia

Leukemia is characterized by abnormal excessive proliferation of hematopoietic stem cells, which can be divided into acute or chronic leukemia.^400–405^ Tp92 is the only outer membrane protein of T. pallidum which has high homology with the outer membrane proteins of many Gram-negative bacteria.^406^ Luo et al. found that the recombinant Tp92 protein was able to cause pyroptosis of THP‐1 cells (the human monocytic cell line derived from an acute monocytic leukemia patient) via the caspase‐1 pathway.^407^ As an effective calcium antagonist, magnesium sulfate (MgSO_4_) can significantly inhibit the release of calcium-influx dependent histamine, and has a potential anti-inflammatory effect.^408–411^ Studies have found that MgSO_4_ can also inhibit LPS-induced increase of NLRP3 inflammasome, upregulation of IL-1β and pyroptosis in THP-1 cells.^412^ CARD8, is a protein encoded by the human genome, consisting of a FIIND followed by a CARD.^413^ The CARD of CARD8 can bind to pro-caspase-1, inducing activation of caspase-1.^414,415^ In general, anti-cancer drug talabostat (PT-100, Val-boroPro) inhibits DPP8/9 (two intracellular serine dipeptidases Dpp8 and Dpp9),^162^ and mainly induces pyroptosis dependent on NLRP1b, but not on ASC.^162,416–418^ Surprisingly, talabostat induces CARD8-mediated pyroptosis in THP-1 and other AML cells, which is independent of NLRP1, indicating that CARD8 can also form inflammasome.^413,419,420^ It has been found that DPP8/9 inhibitors induce pyroptosis in the vast majority of human acute myeloid leukemia (AML) cell lines, and these inhibitors suppress the progression of human AML in mouse models.^162,413^ What’s more, CARD8 was involved in the pyroptosis of human myeloid cells induced by DPP8/9 inhibitors, which provided a new potential strategy for the treatment of AML.^413^ Genetic modification enhanced the ability of chimeric antigen receptor (CAR) T cells to kill target tumor cells.^421–423^ Interestingly, CAR T cells activate GSDME-mediated cell death in B leukemic cells by releasing a large amount of perforin and GzmB, resulting in elevated levels of pro-inflammatory cytokines such as IL-1β, IL-6 and increased release of serum amyloid protein A3 (SAA3), leading to cytokine release syndrome (CRS) in CAR T cell-treated patients.^194^ GSDME can be cleaved by caspase-3 to induce programmed cell death and is considered to be a tumor suppressor gene. However, the high expression of GSDME in B leukemic cells suggests that GSDME may play different role in pore formation of tumor cells.^194^ It means that transforming target tumor cell death mode from pyroptosis to apoptosis may provide a new perspective for the reduction of CRS caused by CAR T cell therapy. Besides, pyridoxine (vitamin B6), a naturally small molecule with low toxicity, induced GSDME-mediated pyroptosis in THP-1 cells, suggesting that pyridoxine is a potential drug for the treatment of AML.^424^

## Therapeutic implications

Different forms of pyroptosis are closely related to tumors.^109^ Currently, researchers have been trying to improve the therapeutic efficacy through pyroptosis or combining pyroptosis with a variety of tumor treatment methods.

### Gasdermin-related therapy

**Fig. 4:**
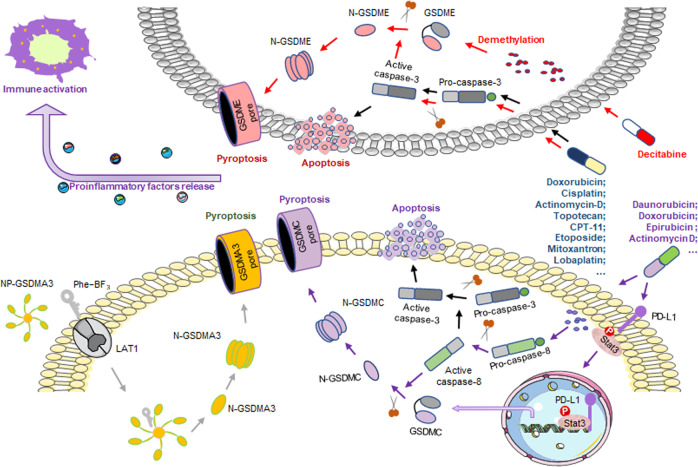
Current status of gasdermin-mediated tumor therapy. The desilylation of Phe-BF3 releases gasdermin from NP-GSDMA3 and induces pyroptosis. A bioorthogonal chemical system was developed in which Phe-BF3 can enter cells through LAT1 (a Phe-BF3 transporter), desilylate and cleave the design linker containing a silyl ether. The purified GSDMA3 (N + C) is conjugated with nanoparticles to become NP-GSDMA3. When Phe-BF3 binds to nanoparticles, the desilylation catalyzed by Phe-BF3 can release active gasdermin from the conjugated nanoparticles and selectively release active gasdermin. Chemotherapeutic drugs induce caspase-3-mediated pyroptosis in tumor cells with high GSDME expression and apoptosis in tumor cells with low expression of GSDME. Decitabine is an inhibitor of DNA methyltransferase, which can demethylate GSDME, changing GSDME expression from low to high, and shift apoptosis to pyroptosis. Almost all chemotherapeutic drugs can induce PD-L1 into the nucleus and promote the expression of GSDMC, but only the antibiotics such as daunorubicin, doxorubicin, epirubicin, and actinomycin D can also activate caspase-8, cause GSDMC cleavage and trigger pyroptosis. Pyroptosis is accompanied by the release of cytokines, which mediate the immune response

**Fig. 5:**
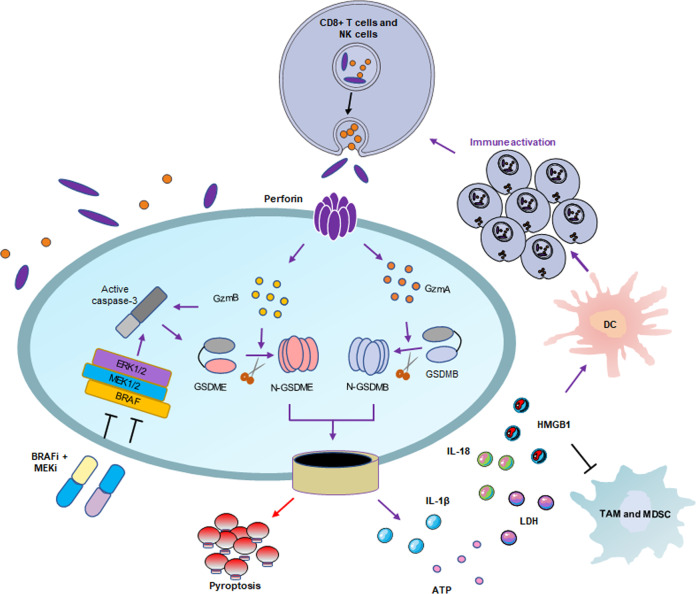
Positive feedback loops of pyroptosis and immune response. After the occurrence of pyroptosis, the formed pores release inflammatory factors, such as Il-1β, HMGB1 LDH, and ATP, which act as alarm signals to activate and recruit immune cells and mediate the immune response. BRAF + MEK inhibitors induce GSDME-dependent pyroptosis mediated by caspase-3, and cause an increase in CD4+ T cell and CD8+ T cell infiltration and a decrease in MDSC and TAM. Cytotoxic lymphocytes release perforin and granzyme, and perforin forms pores in tumor cells. Granzyme enters tumor cells through these pores. GzmA cleaves GSDMB, and GzmB cleaves GSDME, further inducing pyroptosis. This forms a positive feedback loop, which means that a small number of cancer cells undergoing pyroptosis can trigger a tumor immune response and expand the death response

Gasdermins are the effectors of pyroptosis, and activation of gasdermins might prevent tumor formation. The researchers developed a bioorthogonal chemical system mediated by the probe phenylalanine trifluoroborate (Phe-BF_3_, a cancer-imaging probe that is significantly and specifically absorbed by tumors) at the cellular and in vivo levels.^425^ The study revealed that the pyroptosis of a small percentage of tumor cells can effectively regulate the tumor immune microenvironment and activate a strong T cell-mediated antitumor immune response.^425^ This discovery provides a new strategy for the development of tumor immunotherapy drugs (Fig. 4). Wang et al. and Rogers et al. found that chemotherapy drugs induced the activation of caspase-3 in tumor cells with high GSDME expression and induced pyroptosis.^16,17^ In tumor cell lines with low expression of GSDME, decitabine can upregulate GSDME expression and increase the sensitivity of tumor cells to chemotherapy drugs, thus making these cells more prone to pyroptosis^16,17^ (Fig. 4). Lobaplatin-induced ROS led to JNK phosphorylation, and activated JNK promoted the mitochondrial translocation of Bax, which activated GSDME and induced pyroptosis.^281^ This study further elucidated the relationship between chemotherapeutic drugs and pyroptosis. Chen et al. found that PTX-triggered pyroptosis of A549 cells induced was closely associated with activated caspase-3 and N-GSDME levels. Compared with PTX, cisplatin induced more severe pyroptosis in non-small cell lung carcinoma and esophageal squamous cell carcinoma cells, suggesting that cisplatin may have more advantages than other drugs in treating cancer with high expression of GSDME.^362,426^ In addition, the expression of GSDME can enhance the tumor cell phagocytosis by tumor-associated macrophages and increase the number and function of tumor-infiltrating natural killer lymphocytes and CD8+ T lymphocytes. Therefore, the tumor suppressor protein GSDME can enhance immune responses to tumors such as triple-negative breast cancer by activating pyroptosis.^19^ Many different chemotherapy drugs could facilitate nuclear PD-L1 translocation and promote GSDMC expression, but only the antibiotic type drugs was observed to activate caspase-8, and further activated GSDMC activation, resulting in pyroptosis ultimately.^21^ However, this process was abolished by the PD-L1-NLS/Stat3-Y705F mutations or caspase-8 knockout.^21^ Thus, therapeutic methods mediated by this type of drug may cause a strong inflammatory response, thereby affecting the antitumor immunity and prognosis of PD-L1+ or GSDMC+ tumors^21^ (Fig. 4). The combination of BRAF inhibitor and MEK inhibitor led to persistent melanoma regression through immunomodulation.^201^ The combination of these two inhibitors induced the caspase-3 dependent GSDME lysis and the release of pro-inflammatory factors such as HMGB1.^201^ HMGB1 activates dendritic cells by interacting with toll-like receptor 4, thereby promoting anti-tumor T cell activity. Besides, during BRAF inhibitor +MEK inhibitor treatment, both TAM and MDSC decreased.^201^ In addition, immune cells release GzmA and GzmB, which cleave GSDMB and GSDME, respectively, and promote pyroptosis^19,20^ (Fig. 5). Pyroptosis is accompanied by the release of cytokines, which activate the immune response,^23^ indicating that there is a positive feedback regulatory relationship between pyroptosis and the immune response (Fig. 5). In view of the strong antitumor activity of this positive feedback relationship, finding the factors that trigger positive feedback or exploring the relevant regulatory pathways may provide new drug targets for antitumor therapy. Gasdermin family proteins are also potential biomarkers of tumor immunotherapy. The next step may be to explore therapeutic strategies to upregulate gasdermin levels, such as using the existing DNA methylation inhibitor decitabine, which has been approved for the treatment of leukemia and myelodysplastic disorders. The agonists of these proteins are likely to become new candidates in antitumor drug research and development, although the efficacy of these new drugs in tumors remains to be determined.

Interestingly, the inhibition of gasdermins can also effectively block tumor formation. In 2019, Ángela et al. found a new targeted nanomedicine that can deliver a special anti-GSDMB antibody to HER2-positive breast cancer cells, based on hyaluronic acid–biocompatible nanocapsules. This intracellularly-delivered anti-GSDMB antibody effectively and specifically reduced GSDMB functions, thus providing a novel therapeutic strategy for HER2-positive breast cancers.^427^ The high expression of GSDMD was associated with tumor invasion and affected tumor size and stage. In addition, the high expression of GSDMD in lung adenocarcinoma indicated a poor prognosis.^359^ Gsdmd silencing in NSCLC cells decreased EGFR-AKT signaling, enhanced caspase-3 cleavage and apoptosis, and inhibited transplanted tumor growth in NSCLC cells in mice. Gsdmd silencing also reduced EGFR-AKT signaling, enhanced caspase-3 activation and apoptosis, and inhibited transplanted tumor growth in mice.^359^ These findings indicate that inhibiting gasdermins may also be a potential strategy for tumor therapy.

### Inflammasome-related therapy

Inflammasomes are cytoplasmic polyprotein complexes.^142^ In addition, activated caspase-1 cleaves GSDMD, leading to pyroptosis.^428^ Inflammasomes take part in diverse hallmarks of tumor formation and progression, promoting and inhibiting tumors.^429–431^

**Fig. 6:**
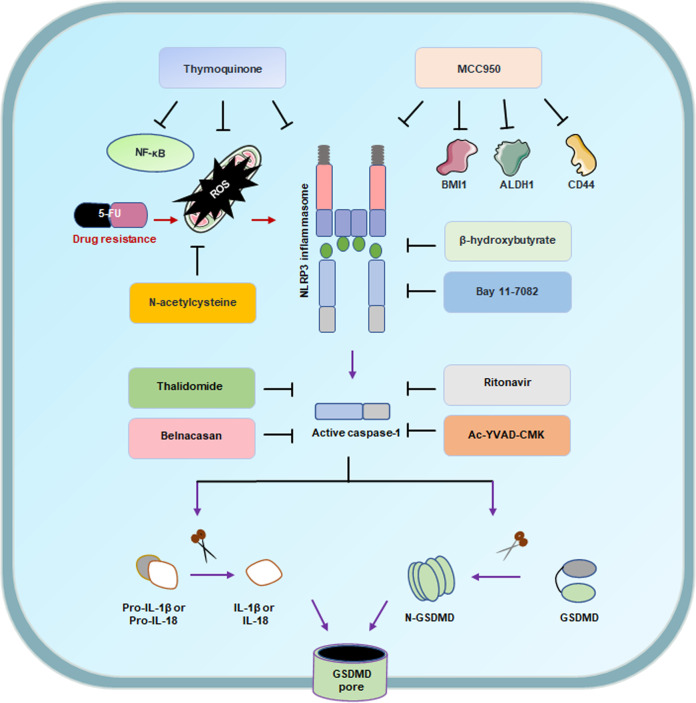
The status of current NLRP3 inflammasome and caspase-1 inhibitors

Initially, inflammasomes promote tumorigenesis, and so the inhibition of inflammasomes may provide a promising tumor treatment. The lack of NLRP3 significantly reduced lung metastasis and improved the survival rate of melanoma in response to dendritic cell therapy. The depletion of NLRP3 resulted in a great decrease in the number of MDSCs, thus inhibiting the activity of neighboring immune cells.^220^ The thymoquinone is the main component extracted from the black seeds of *Nigella sativa*, and it inhibited the migration of melanoma cells by inhibiting the NLRP3 inflammasome.^432^ MCC950, an NLRP3 inhibitor, can reduce the number of spheres and colonies formed by SCCHN and suppress the expression of BMI1, ALDH1, and CD44 in SCCHN cells.^433^ The specific NLRP3 inflammasome blocker β-hydroxybutyrate has also shown promise as a treatment in inflammasome-associated fields.^434^ In addition, the expression of NLRP3 in tumor-infiltrating macrophages was associated with survival, lymph node invasion, and metastasis in patients with mammary cancer.^435^ The NLRP3 inflammasome promoted the drug resistance of oral squamous cell carcinoma (OSCC) to 5-fluorouracil (5-FU). Targeting the ROS/NLRP3 inflammasome/IL-1β signaling pathway may be helpful for 5-FU adjuvant chemotherapy in OSCC.^436^ Bay 11–7082 can inhibit the NLRP3 inflammasome,^437^ blocking the cell cycle progression of gastric cancer cells, which provides a basis for its clinical application in gastric cancer.^438^ The expression of ASC protein in metastatic melanoma is lower than that in primary melanoma. In primary melanoma, ASC inhibits tumorigenesis by reducing the phosphorylation of IKKalpha/beta and inhibiting the activity of NF-kappaB.^212^ ASC has a pro-inflammatory effect on infiltrating cells, which is beneficial to the development of melanoma, but it also limits the proliferation of keratinocytes to respond to harmful stimuli, possibly by activating p53 to inhibit melanoma.^439^ In addition, antioxidants inhibit the production of ROS, suggesting that they block the expression of NLRP3. N-acetylcysteine (NAC) is a common antioxidant that blocks the activation of the NLRP3 inflammasome in patients.^440^ NLRP3 and AIM2 were overexpressed in EBV-associated NPC, and the expression level was related to patient survival.^441^ The exact effects of NLRP3 in different tumors also highlights the therapeutic potential of the inflammasome as a prognostic marker. Knockdown of AIM2 resulted in the inhibition of cell growth and apoptosis in OSCC cells, while B cells activation was downregulated by nuclear factor kappa-light-chain enhancement.^442^ Furthermore, the NLRC4 inflammasome activation/IL-1a signaling pathway, which is associated with obesity, promoted the progression of breast cancer, which provided a mechanism by which obesity can promote the progression of breast cancer. This result may have a certain reference value in the treatment of obese cancer patients.^271^ These findings suggest that inhibiting inflammasomes may be beneficial and safe for the prevention and treatment of tumors (Fig. 6).

In addition, inflammasomes also have antitumor effects, which has been confirmed in a model of colorectal cancer. For example, NLRP1 was significantly dysregulated in colon cancer and attenuated tumorigenesis. Deletion of Nlrp1b significantly increased morbidity and tumorigenesis in mice.^282^ In addition, Nlrp3 (−/−) mice also showed recurrent colitis and increased colitis-associated cancer.^283,284^ Besides, the lack of the NLRP6 inflammasome enhanced inflammation-induced CRC development in mice.^443^ In DSS-induced colitis, pyrin is necessary to activate the inflammasome and mature IL-18.^287^ Pyrin can also promote the integrity of the intestinal barrier and prevent colitis and tumors.^287^ In summary, strategies to improve the activity of inflammasomes may be developed to prevent the occurrence of related tumors.

### Caspase 1-related therapy

Caspase-1, as an upstream effector, is also crucial in pyroptosis. Thalidomide is an effective anti-inflammatory drug that significantly inhibits the activation and activity of caspase-1, but its application is limited because of its strong teratogenic activity.^444^ When T cells were depleted, the growth rates of tumors in chimeric mice with adoptive transfer of caspase-1-deficient bone marrow cells were significantly inhibited.^445^ This antitumor response was reversed by wild-type MDSCs in adoptive metastatic tumor-bearing mice, while the antitumor effect of caspase-1 on MDSCs treated with thalidomide was similar to that in caspase-1-deficient mice.^445^ Belnacasan (VX-765) is an effective, selective caspase-1 inhibitor. However, its clinical effect has not been optimistic because while it is highly tolerated, preclinical data showed that its curative effect was lower than expected.^446^ In addition, VX-765 effectively suppressed the production of inflammatory factors, reduced the inflammatory response, and protected the gastric mucosa of mice with acute gastric ulcers induced by cold-restraint stress.^447^ Ritonavir, once a protease inhibitor used to treat HIV, was later found to effectively reduce the level of IL-18 in mouse pancreatic cancer by suppressing caspase-1.^448^ Ac-YVAD-CMK, an irreversible and selective inhibitor of caspase-1, effectively blocked the cleavage of pro-IL-1β and pro-IL-18 and reduced the inflammatory response and is expected to be used to inhibit the progression of esophagitis.^449^ Furthermore, Ac-YVAD-CMK suppressed IFI16- or berberine-induced tumor inhibition in hepatocellular carcinoma ^335,450^ (Fig. 6).

## The negative effects of pyroptosis on tumor therapy

Previous studies have shown that the activation of GSDME is caspase-3-dependent, and the expression level of GSDME is closely associated with the mode of cell death induced by chemotherapeutic drugs. Chemotherapeutic drugs induced pyroptosis in cells with high expression of GSDME, while cells with low or no expression underwent apoptosis. However, the researchers also pointed out that GSDME expression was low in most tumor cell lines due to GSDME gene promoter methylation, while GSDME was widely overexpressed in normal cell lines.^16,17^ Therefore, chemotherapy could also induce caspase-3-mediated pyroptosis in normal cells with high expression of GSDME, which may be one of the causes of toxicity and side effects associated with chemotherapy. In Gsdme+/+ mice, intraperitoneal injection of cisplatin or 5-FU caused immune cell infiltration and severe small intestinal injury, while the signs of tissue damage decreased significantly in Gsdme−/− mice.^451^ In addition, Gsdme−/− mice exhibited less inflammation and lung injury under bleomycin or cisplatin treatment.^451^

Pyroptosis is also involved in the negative effects of radiotherapy. The AIM2 inflammasome played an unexpected role in responding to radiation-induced DNA damage and induced caspase-1-mediated pyroptosis in intestinal epithelial cells and bone marrow cells, which is one of the causes of radiation-induced gastrointestinal and hematological toxicity.^452^ Inhibition of the AIM2 inflammasome may be a promising treatment target for some patients, such as those exposed to ionizing radiation or cancer patients suffering from hematopoiesis or gastrointestinal toxicity due to radiotherapy or chemotherapy. In addition, radiation also triggered NLRP3 inflammasome-mediated pyroptosis in bone marrow-derived macrophages.^453^ Targeting the NLRP3 inflammasome may be an effective strategy to reduce radiation damage. In addition, flagellin A N/C inhibited radiation-induced ROS production in intestinal epithelial cells, reduced NLRP3 activity, and suppressed caspase-1-dependent pyroptosis, which may be a factor in protecting intestinal epithelial cells from radiation damage.^454^ These studies show the negative effects of pyroptosis in chemotherapy and radiotherapy, which offer new insight and hope to tumor treatments.

## Conclusion

As an inflammatory and programmed mode of cell death, pyroptosis has been gradually elucidated with further research, but there are still some problems to be solved, such as what other factors regulate pyroptosis and what role other members of the gasdermin family play in pyroptosis.

As described above, increasing evidence has confirmed the important role of pyroptosis in tumors, but few tumor-specific regulatory mechanisms have been identified. Moreover, the conclusion about the relationship between pyroptosis and tumors is not entirely consistent, which indicates the heterogeneity of tumors and the complexity of the immune microenvironment. Pyroptosis can not only inhibit tumor cell proliferation, but also form a microenvironment suitable for tumor cell growth and promote tumor growth. Currently, chronic tumor necrosis caused by pyroptotic cell death of a small number of tumor cells in the central hypoxic area of the tumor inhibits antitumor immunity and accelerates tumor development. The acute inflammation induced by pyroptosis in the tumor microenvironment enhances the immune response and suppresses the progression of the tumor. However, the dual mechanism of pyroptosis related factors promoting and inhibiting tumorgenesis and development remains to be explored. What’s more, the roles of pyroptosis agonists and inhibitors in tumor microenvironment are worthy of further clinical study.

Although there are still many gaps in the knowledge of the role of pyroptosis in tumors, further study on the signaling pathway, regulatory mechanism, and pathological significance of pyroptosis will be helpful in developing new therapeutic options for preventing and treating human tumors.

In addition to the well-studied chemotherapeutic drugs and some small molecular drugs, the therapeutic response to pyroptosis and radiotherapy and the relationship between pyroptosis and specific immunotherapy are not completely clear. Pyroptosis pathway agonists can be used as antitumor activators, and tumor regression can be realized depending on subsequent immune amplification (Fig. 5). The gasdermin proteins do not need to kill all cancer cells but activate tumor immunity by killing a small number of cancer cells. It is difficult to avoid damaging some normal tissue cells. However, since immune cells are able to distinguish normal tissue cells from tumor cells, pyroptosis-mediated immune amplification will selectively occur in tumor cells, greatly reducing the toxic and side effects of drugs on normal tissues. It is possible for researchers to find some small molecules that bind to gasdermin to alleviate the self-inhibition of gasdermin and induce pyroptosis. If such small molecules can be used in clinical treatment by oral or intravenous administration, doctors can accurately control the dose and ensure drug safety. Unfortunately, at present, the regulatory effect of gasdermin family proteins on tumors is not very clear and may be used as tumor-promoting molecules or as tumor suppressors.

We cannot accurately explain whether gasdermin agonists or inhibitors should be used to achieve tumor inhibition. Further elucidation of the important role of gasdermin in the tumor immune response is needed, which will also provide possible strategies for precision treatment of tumor immunity. Specifically, since immune cells can kill cancer cells by inducing cell death,^19,20^ gasdermin family proteins may be used as biomarkers to divide patients into clinical groups. Patients with high expression of gasdermin family proteins may have improved responses to tumor immunotherapy.

What is the effect of chemotherapeutic drugs on the tumor immune microenvironment by activating GSDME and causing pyroptosis in normal cells with high GSDME expression? How to regulate tumor cell pyroptosis to achieve the desired therapeutic effect and how to avoid normal cell pyroptosis during tumor treatment still need to be explored. It is expected that basic research on pyroptosis and tumors will continue to improve and translate to clinical practice.
